# Development of CBCT-based prostate setup correction strategies and impact of rectal distension

**DOI:** 10.1186/s13014-015-0386-8

**Published:** 2015-04-10

**Authors:** Christine Boydev, Abdelmalik Taleb-Ahmed, Foued Derraz, Laurent Peyrodie, Jean-Philippe Thiran, David Pasquier

**Affiliations:** Signal Processing Laboratory, Swiss Federal Institute of Technology Lausanne (EPFL), Lausanne, Switzerland; Laboratory of Industrial and Human Automation control, Mechanical engineering and Computer Science, University of Valenciennes and Hainaut-Cambrésis, Valenciennes, France; Unité de Traitements de Signaux Biomédicaux, Faculté Libre de Médecine, Lille, France; Unité de Traitements de Signaux Biomédicaux, Hautes Etudes d’Ingénieur, Lille, France; Department of Radiology, University Hospital Center (CHUV) and University of Lausanne, Lausanne, Switzerland; Academic Department of Radiation Oncology, Centre Oscar Lambret, Lille, France

**Keywords:** Image registration, Cone-beam computed tomography, Image-guided radiotherapy, Prostate cancer

## Abstract

**Background:**

Cone-beam computed tomography (CBCT) image-guided radiotherapy (IGRT) systems are widely used tools to verify and correct the target position before each fraction, allowing to maximize treatment accuracy and precision. In this study, we evaluate automatic three-dimensional intensity-based rigid registration (RR) methods for prostate setup correction using CBCT scans and study the impact of rectal distension on registration quality.

**Methods:**

We retrospectively analyzed 115 CBCT scans of 10 prostate patients. CT-to-CBCT registration was performed using (a) global RR, (b) bony RR, or (c) bony RR refined by a local prostate RR using the CT clinical target volume (CTV) expanded with 1-to-20-mm varying margins. After propagation of the manual CT contours, automatic CBCT contours were generated. For evaluation, a radiation oncologist manually delineated the CTV on the CBCT scans. The propagated and manual CBCT contours were compared using the Dice similarity and a measure based on the bidirectional local distance (BLD). We also conducted a blind visual assessment of the quality of the propagated segmentations. Moreover, we automatically quantified rectal distension between the CT and CBCT scans without using the manual CBCT contours and we investigated its correlation with the registration failures. To improve the registration quality, the air in the rectum was replaced with soft tissue using a filter. The results with and without filtering were compared.

**Results:**

The statistical analysis of the Dice coefficients and the BLD values resulted in highly significant differences (p<10^−6^) for the 5-mm and 8-mm local RRs vs the global, bony and 1-mm local RRs. The 8-mm local RR provided the best compromise between accuracy and robustness (Dice median of 0.814 and 97% of success with filtering the air in the rectum). We observed that all failures were due to high rectal distension. Moreover, the visual assessment confirmed the superiority of the 8-mm local RR over the bony RR.

**Conclusion:**

The most successful CT-to-CBCT RR method proved to be the 8-mm local RR. We have shown the correlation between its registration failures and rectal distension. Furthermore, we have provided a simple (easily applicable in routine) and automatic method to quantify rectal distension and to predict registration failure using only the manual CT contours.

## Background

Many studies have demonstrated that dose escalation increases local tumor control with acceptable toxicity [1,2]. With intensity-modulated radiotherapy (IMRT), it has become possible to deliver higher doses to the target and reduce the dose to the surrounding normal tissue. However, internal organ motion can occur over the course of radiotherapy and cause an underdosage of the target and an overdosage of the organs at risk (OAR). To compensate for target mobility, population-based margins are added to ensure proper dose coverage of the target [3]. This in return may increase toxicity to neighboring normal tissue. Daily image guidance makes it possible to reduce these treatment margins and organ toxicity by helping to provide a precise knowledge of the actual position of the target at treatment [2,4]. In the context of prostate cancer radiotherapy, daily image-guidance is particularly useful. Indeed, the prostate gland is known to be a moving and deformable gland, which can be influenced by changes in rectal and bladder volumes [5-7]. It should be noted that bladder filling has a substantially smaller influence on prostate motion than rectal distension has [8].

There have been many efforts to localize the prostate for accurate delivery before daily treatment, including transabdominal ultrasound imaging [9], kilovoltage or megavoltage orthogonal port films of implanted gold fiducial markers [10], portal images of a urethral catheter containing radioopaque markers [11] and electromagnetic tracking devices [12]. More recently, in-room tomography imaging devices have gained attention and have become commonplace in clinical centers. A great number of authors used registration with the planning computed tomography (CT) scan to localize the prostate on the day of the treatment. Most studies were performed using daily in-room CT imaging systems as in [13-17]. Cone-beam computed tomography (CBCT) image-guided radiotherapy (IGRT) systems [18] have become widely used tools for prostate positioning in IMRT. However, due to the much poorer image quality of CBCT scans than that of CT scans, prostate localization on CBCT scans is more challenging [19]. Daily in-room CBCT imaging for prostate cancer was used in [20-29] but few studies have localized the prostate in a completely automated way.

This paper aims to evaluate different automatic registration methods for the purpose of prostate position verification and correction using CBCT imaging. It is commonly assumed that the prostate gland behaves as a rigid body [8] and that the deformation of the prostate during the course of radiotherapy is small compared to the organ motion [30]. Consequently, during IGRT of prostate cancer, in first order approximation, only set-up error and organ motion need to be corrected, whereas prostate deformation can be considered to be a second-order effect. That is why we focused on rigid registration (RR), which accounts for first-order inter-fraction prostate motion. In this work, we tested different types of CT/CBCT RRs: global, bony, and local soft-tissue RRs.

Unlike previous studies whose quantitative validation consisted of estimating CT/CBCT registration errors at landmark positions (e.g., fiducial markers or calcifications), our study contains a quantitative validation based on Dice calculations which provide a global estimation of the registration accuracy at the location of the target. By definition, the Dice coefficient quantifies the overlap between regions, and in this work we used it to measure the mismatch between the (manual and automatic CBCT) prostate volumes.

We also defined a practical method to automatically estimate rectal distension occurred between the CT and CBCT scans in order to evaluate the impact of rectal distension on the registration quality and to predict registration failure using only the CT manual contours and the gray-value CT and CBCT images. For this purpose, we based our reasoning on the fact that the variation in the volume of air in the rectum was directly correlated with rectal distension. To the best of our knowledge, no publications to date have presented a simple automatic method to quantify rectal distension occurred between the X-ray CT and CBCT scans using only the manual CT contours and the gray-value CT and CBCT scans. Finally, we devised recommendations for clinical practice for the use of automatic RR for prostate localization on CBCT scans.

In this paper, we used the terms clinical target volume (CTV) and planning target volume (PTV) as defined by the ICRU [31].

## Methods

### Data description

In total, 115 CBCT scans from 10 prostate cancer patients were analyzed. Each one of these patients underwent a single planning CT scan and multiple daily CBCT scans over the course of treatment. All the patients were instructed to follow a dietary protocol in order to have a full bladder and an empty rectum free of air at the time of the planning CT acquisition and during the treatment. The planning CT data were acquired using a General Electrics Light Speed scanner. The treatment system was an ELEKTA Synergy linear accelerator (LINAC) equipped with CBCT imaging (named x-ray volume imaging (XVI)). During the CT (CBCT) acquisition, the peak-voltage, the X-ray tube current and the exposure time were 120 kVp (120 kVp), 300 mA (40 mA or 64 mA) and 1000 ms (40 ms), respectively. Combined with the difference in beam geometry (fan for CT and cone for CBCT), these parameters accounted for the lower image quality obtained with CBCT as compared with CT. The slice thickness was 3 mm and 1 mm for the CT and CBCT scans, respectively. The number of slices ranged from 89 to 132 in each CT scan, and was 168 in each CBCT scan. Each CT (CBCT) slice had 512 × 512 (410 × 410) pixels, with a typical in-plane resolution of 0.98 (1.00) mm.

For clinical requirements, the prostate CTV (without seminal vesicles) and the OAR, i.e., the bladder and the rectum, were manually delineated on each planning CT scan by a radiation oncologist. For the purposes of this study, the same radiation oncologist manually delineated the CTV and the OAR on each CBCT scan, following the consensus contouring guidelines provided by the Radiation Therapy Oncology Group at www.rtog.org/LinkClick.aspx?fileticket=054g99vNGps%3d&tabid=354. The bladder was contoured in its entirety. The rectum was contoured from the rectosigmoid junction to the anal verge. This anonymized database (composed of the anonymized gray-value CT and CBCT images with their manual segmentations) is now available by request via email to the authors.

### Registration algorithm

**Figure 1:**
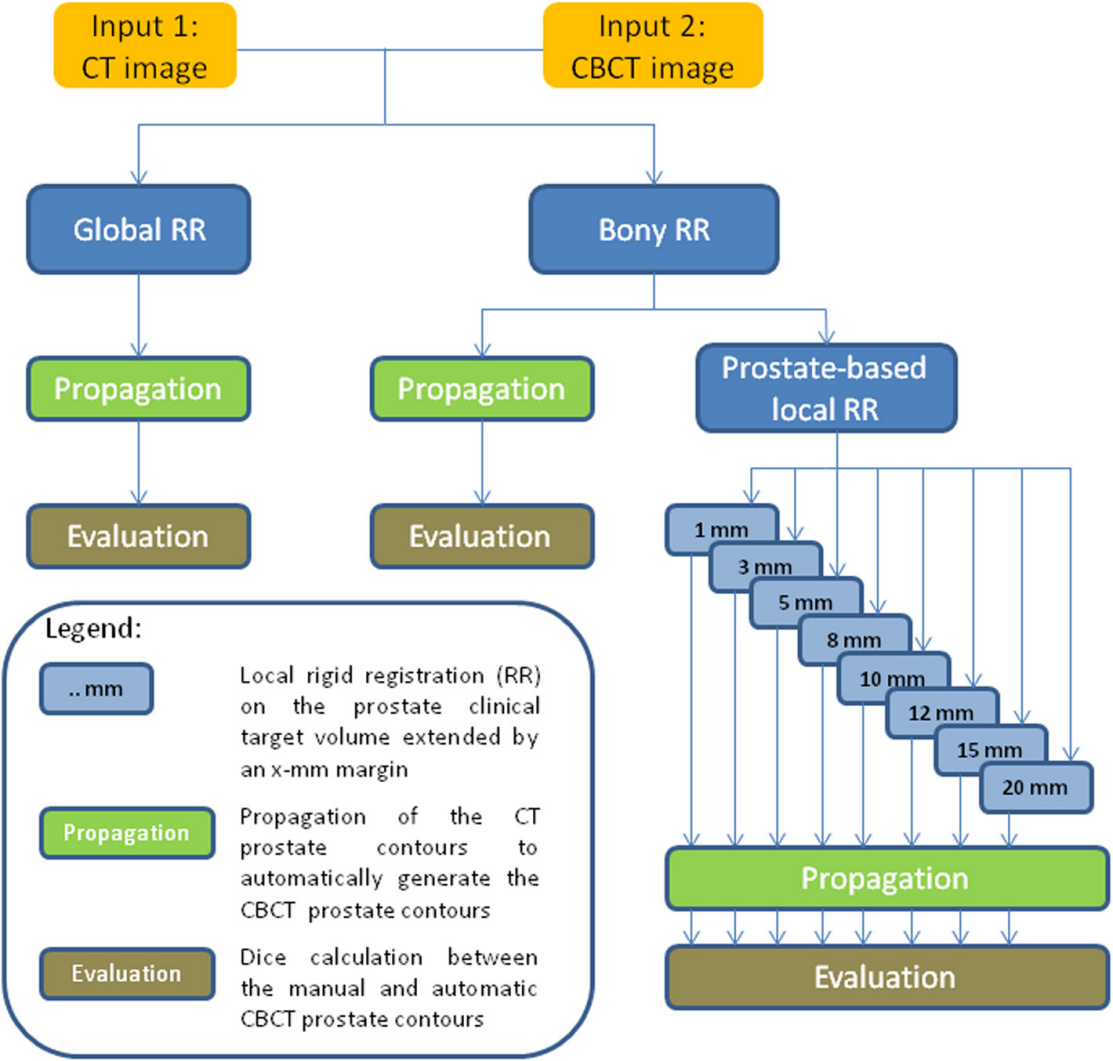
**Registration pipeline.**

To automatically localize the prostate on the 115 daily treatment CBCT scans, each of them was registered to the corresponding planning CT scan using different registration methods described hereunder. The resulting displacements were then applied to the contours drawn manually on the planning CT scans to generate the automatic propagated CBCT contours. The skeleton of our 3D automatic intensity-based registration procedure was previously described in [32]. To overcome the problem of the variable amount of fecal gas in the rectum which could mislead registration, we performed an extra pre-processing filtering step which replaced gray values of gas by a tissue-equivalent gray value as recommended in [17] in addition to our pipeline described in [32]. We used a threshold gray value of -150 HU for the CT scans and -500 for the CBCT scans (all gray values below were set to these threshold values). We compared these results with those obtained without filtering. In our study, three types of intensity-based RR methods were tested (Figure 1):

- global RR,
- RR of the pelvic bone structures of the CT and CBCT images (bony RR),
- bony RR followed by a local soft-tissue RR based on target (prostate) information. The latter was conducted using a registration mask that was a region of interest (ROI) defined by the CT CTV expanded with a margin among 1, 3, 5, 8, 10, 12, 15 and 20 mm. The CTV represented the whole prostate gland, which was manually delineated in the (clinical) planning process prior to treatment.

In the following, for the sake of simplicity, the combination of a bony RR with a local soft-tissue RR (method (c)) is referred to as a local RR.

A typical image registration framework has four basic components: a similarity metric, an optimizer, a transform and an interpolator. The similarity metric (or metric) measures quantitatively how well a transform is mapping the reference image on top of the target image. We used an intensity-based metric, which allowed the registration to be fully automatic. A simple metric such as mean squared differences could not be used as it required that the images to be registered should have intensity values in the same range, i.e., be monomodal images. In fact, the context was not strictly monomodal image registration since the CBCT system was not calibrated in Hounsfield units. However, the relationship between the intensities on the CT image and those on the CBCT image was given by a linear function. The normalized cross-correlation metric was therefore chosen as a suitable similarity metric (with mean intensities subtracted). This function computed the correlation between the intensity values divided by the square rooted autocorrelation of both the target and reference images. We performed a (deterministic) gradient descent optimization, which was the most straightforward method for incorporating gradient information into the minimization process. The optimizer simply followed the derivative of the metric. At each iteration, the current position was updated according to the gradient of the metric multiplied by a learning factor defined as a step size multiplied by a relaxation factor every time that the gradient changed direction. We used a constant step size of 0.5, a step size relaxation of 0.7, a tolerance on the step size of 0.1, a tolerance on the projected gradient magnitude of 10^-5^ and a maximum number of iterations of 500. Transformations were rigid and hence they had only six degrees of freedom (translations and rotations). Linear interpolation was used in all our experiments. A multi-resolution registration scheme, using three resolution levels, was also utilized. Notice that when masks were associated with the images to be registered, only pixels that belonged to the intersection of the masks were considered for the computation of the metric.

**Figure 2:**
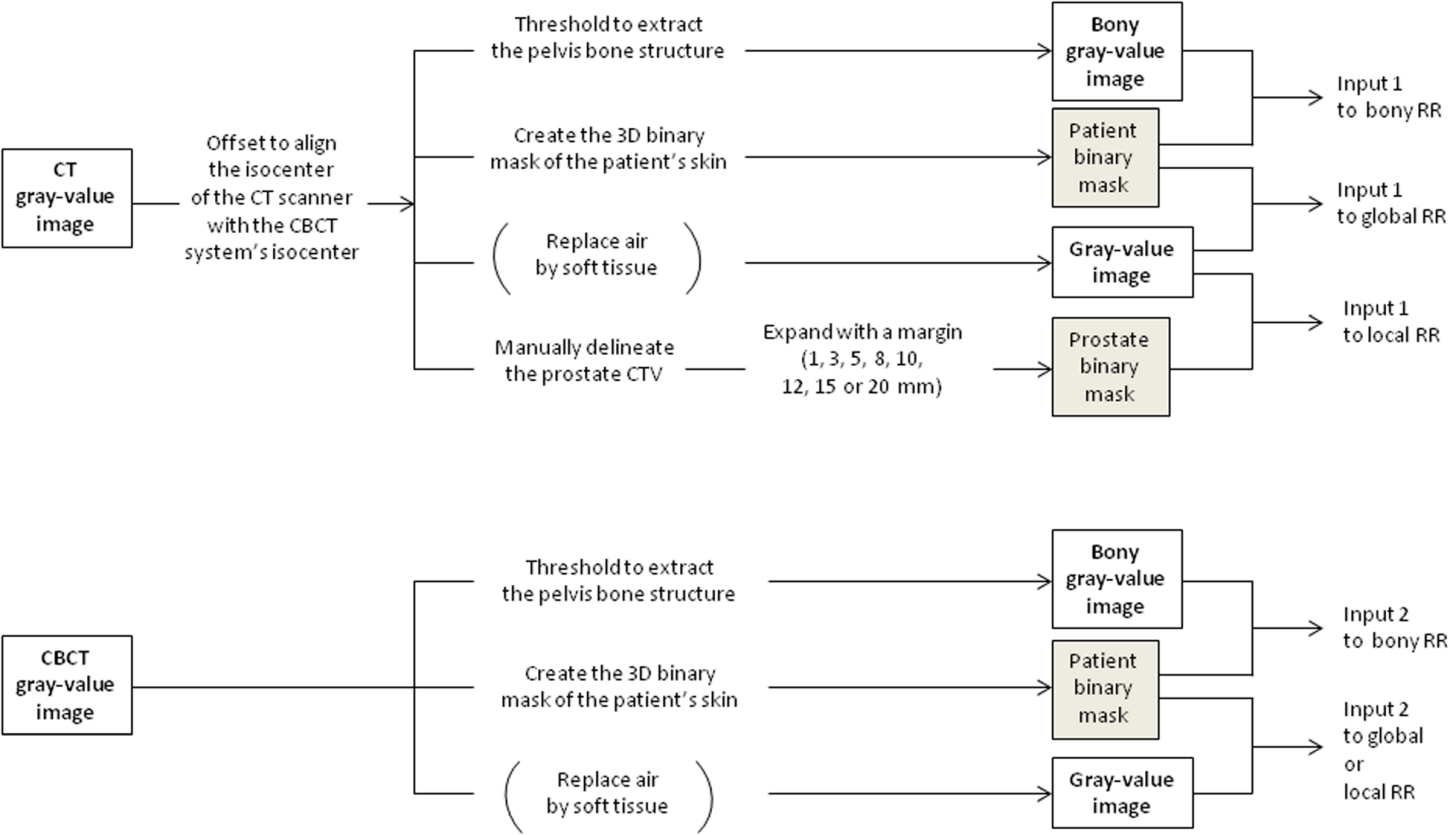
**Pre**-**processing pipeline.**

Prior to CT/CBCT RR, there were some pre-processing steps to carry out (Figure 2):

1. We offset the planning CT image so that its isocenter coincided with the treatment isocenter (i.e., the isocenter of the CBCT system). To do so, the information concerning the planning CT isocenter coordinates was retrieved from the Dicom RTStruct file and compared with the LINAC isocenter coordinates (set to 0,0,0).
2. We created masks that identified the patient’s body on both gray-value images. These were the masks associated with the gray-value images by default (if no other masks were used) to allow registration to ignore pixels that were “outside” the patient (e.g., the treatment table or artifacts) and could adversely influence the registration process.
3. *(This step concerned the bony RR only.)* We thresholded the CT and CBCT gray-value images to exclusively show the pelvis bone structure. The threshold level used to extract the bone anatomy was 150 Hounsfield units (HU) for the CT images, and -140 arbitrary units for the CBCT images (in our institution, the CBCT system was not calibrated in HU). These thresholded images were registered, each one associated with its corresponding patient body mask.
4. *(This step concerned the global and the local soft-tissue RRs only.)* We replaced the air in the rectum by a tissue-equivalent gray value in the CT and CBCT images (the registration results were compared with those obtained without this step).
5. *(This step concerned the local soft-tissue RR only.)* We expanded the physician-drawn CT CTV by a margin of 1, 3, 5, 8, 10, 12, 15 or 20 mm. This created the mask to associate with the CT gray-value image in the local soft-tissue RR process, instead of the CT patient body mask created in step 2.

### Software

For this study, all the data processing and visualization were performed on a Linux computer with distribution openSUSE 11.4 ×86 64, with an Intel Dual Core i5-560 M 2.66 GHz processor, 3 MB L2 cache, 4 threads, and 8GB RAM. For the implementation of our registration algorithms, the following open-source software, based on C++, was used:

- the Insight Toolkit ITK [33]^[a]^
- the ITK-based Command Line Image Toolkit clitk^[b]^.

The software versions used were ITK 4.3.2, CMake 2.8.3 and gcc 4.5.1.

### Validation

Software R, version 2.12, was used for all statistical significance testing [34]. A p-value *<* 0*.*05 (*<* 0*.*01) was considered (highly) significant. This part is composed of a quantitative section and a qualitative section. The quantitative section was presented in part at the 2013 International Conference on the Use of Computers in Radiation Therapy [35] and was conducted using fewer scans and patients. The main new contributions here include the following. We conducted a qualitative validation that provided additional information during data analysis. As mentioned above, we also performed an extra pre-processing step that filtered the air in the rectum to improve the registration quality. We also calculated the Dice similarity coefficient between the CT and CBCT manual CTV contours for each CT/CBCT pair after contour RR, corresponding to the maximum value achievable by any intensity-based RR method. Finally, we used an additional measure based on the bidirectional local distance (BLD) to assess the quality of all registration methods.

#### Quantitative validation

##### Dice coefficient

The Dice similarity coefficient was calculated between the manual (ground-truth) and the automatic (propagated) prostate segmentations on each CBCT scan and for each method. The Dice coefficient between a volume, A, and a volume, B, is defined as follows [36]:

1 $$ DiceCoefficient=\frac{2\cdot \left(A\cap B\right)}{A+B} $$

Ideally, when two volumes overlap perfectly, the Dice coefficient equals 1. A null Dice coefficient would correspond to two disjoint volumes. Differences in the Dice results across the multiple intensity-based RR methods were tested for significance using the inferential non-parametric Friedman statistical test (with *α* set to 0.05), a version of the parametric repeated-measures ANOVA. The Wilcoxon-Nemenyi-McDonald-Thompson post-hoc test was conducted to decide which methods were significantly different from each other ([37], page 295).

We assumed that the registration was unsuccessful if the Dice coefficient after registration was found to be lower than 95% of the Dice coefficient without registration. Indeed, we decided that below this threshold, the choice of which will be explained in the Results section, performing a registration would deteriorate the initial image alignment (before/without registration). The Dice coefficient without registration was calculated after applying an offset to the planning CT image so that its isocenter coincided with the treatment isocenter (i.e., the isocenter of the CBCT system); this value was calculated between the manual and the automatic prostate segmentations on the CBCT, the automatic one being simply the manual prostate segmentation on the CT scan (no registration was considered and therefore, no propagation applied).

The Dice coefficient was also calculated between the CT and CBCT manual CTV contours for each CT/CBCT pair after contour RR, i.e., after RR of the binary masks of the manual contours. This Dice value represented an upper bound on the Dice coefficients calculated for the intensity-based RR methods., i.e., the maximum value achievable by any intensity-based RR method.

##### Bidirectional distance

To accurately quantify the difference between the manual (ground-truth/reference) and the automatic (propagated) prostate segmentations on each CBCT scan and for each method, we also used the bidirectional local distance (BLD), a robust point-to-surface distance measure introduced by Kim *et al.* in [38]. At each point on the reference contour, a BLD was calculated, and then all BLDs over the reference contour were averaged to obtain a global bidirectional distance (BD) between both contours.

##### Impact of rectal distension on local RR quality

In general, the performance of RR deteriorates when the size or the shape of an organ changes. When performing local RR on the prostate ROI (CTV extended with a margin among 1-20 mm), the registration mask necessarily includes a portion of the rectum, as the prostate is in contact with the rectum. However, the rectum is highly prone to changes in size and shape due to its ever-changing filling (gaseous and solid contents). The hypothesis we wished to validate was that the unsuccessful local RRs were caused by rectal distension occurred in the vicinity of the prostate. We related the successful and unsuccessful local RRs to the difference in rectum filling between the CT and the CBCT scans in the region of the rectum that was included in the registration mask. For this purpose, for each CT/CBCT pair, we calculated the following variable to quantify rectum filling variation, that is rectal distension:

2 $$ F=\left|\left({\overline{I}}_{CBCT,r}-{\overline{I}}_{CBCT,p}\right)-\left({\overline{I}}_{CT,r}-{\overline{I}}_{CT,p}\right)\right| $$

**Figure 3:**
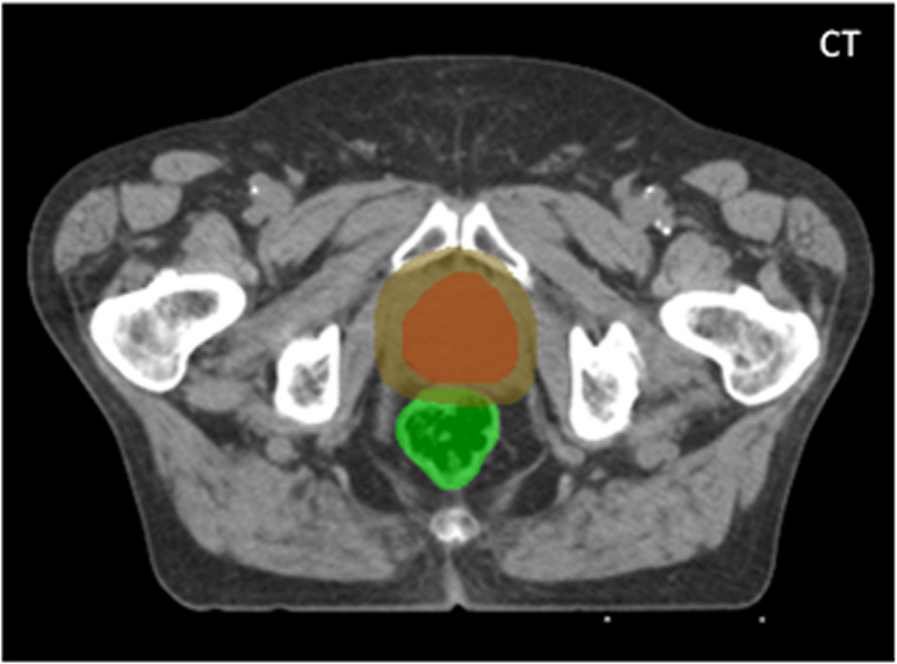
**CT slice.** Slice of a CT scan. The manual contours of (red) the prostate and (green) the rectum, as well as (brown) the ROI defined as the prostate gland extended with a margin of 8 mm are drawn. The rectal distension occurred between the CT and the CBCT scans was estimated by calculating the value of *F* as defined in Equation 2. *Ī*_*CT*,*p*_(*Ī*_*CBCT*,*p*_) was calculated within the red region on the CT (CBCT after bony rigid alignment) scan. *Ī*_*CT*,*r*_(*Ī*_*CBCT*,*r*_) was calculated within the intersection of the green and the brown regions on the CT (CBCT after bony rigid alignment) scan.

Where *Ī*_*CT*,*r*_ is the CT average intensity in the rectum portion, *R*_*partial*_, included in the registration mask, *Ī*_*CBCT*,*r*_ is the CBCT average intensity in region *R*_*partial*_ after rigidly aligning the bony structures of the CT and CBCT scans, *Ī*_*CT*,*p*_ is the CT average intensity in the prostate, and *Ī*_*CBCT*,*p*_ is the CBCT average intensity in the prostate after rigidly aligning the bony structures of the CT and CBCT scans. We used the manual segmentations to generate region *R*_*partial*_, which corresponded to the intersection of the mask and the rectum volume on the CT scan (Figure 3). As the overall range of intensities on a reconstructed CBCT scan could shift across acquisitions, *Ī*_*CBCT*,*p*_ and *Ī*_*CT*,*p*_ were subtracted from *Ī*_*CBCT*,*r*_ and *Ī*_*CT*,*r*_, respectively.

The *F* number given by Equation 2 consistently reflects rectal distension occurred in the vicinity of the prostate. Indeed, in a CT or a CBCT scan, when the rectum is empty (free of air), it is represented by the same range of intensities as the prostate as both the rectum and the prostate are soft tissues. When the same rectum is filled with gas and solid contents, its volume increases and its pixel intensities are shifted to lower values on average.

We plotted the success/failure output from all registrations, arranging the 115 CT/CBCT pairs according to increasing *F* number.

#### Visual assessment

A blind visual assessment of the quality of the propagated segmentations was conducted to confirm the quantitative results. Each propagated prostate segmentation was displayed onto the corresponding CBCT scan and the radiation oncologist was asked to indicate the number of slices that needed to be corrected, without knowing the registration method used. A quality score between 0 and 3 was given to each automatic segmentation as follows: 0 if the quality was poor, 1 if a major deviation could be edited (more than 3 slices needed to be corrected), 2 if a minor deviation could be edited (3 slices or less needed to be corrected), 3 if the quality was perfect (no need to edit any slice). Thus, for our set of 115 CBCT scans, the maximum possible cumulative quality score was 345, whereas a score of 230 would indicate a fair mean performance. The non-parametric Wilcoxon signed-rank statistical test was conducted to evaluate the difference between the bony and the local RR methods. The radiation oncologist also assessed whether the propagated segmentations could be used as such for clinical practice, without further correction.

## Results

### Without applying a replace-gas-by-tissue filter

#### Dice coefficient

We assessed the quality of a registration by comparing the automatic and manual CBCT prostate contours. All manual contours were delineated by the same radiation oncologist. Thus, in the calculation of the Dice coefficient, there was an uncertainty due to the intra-observer and inter-modality variability in manual organ delineation, which we needed to account for in the way we assessed unsuccessful registration. For this purpose, we registered the binary masks of the manual contours of the CT and CBCT images (referred to as contour RR). We found that our set of CT-to-CBCT manual-contour RRs had a Dice mean of $\overline{DSC} = 0.858$ and a SD of 0.035. Approximately 99% of those Dice values lied in interval $\left[\overline{DSC}\hbox{--} 2.58\ast SD,\overline{DSC} + 2.58\ast SD\right]$, being [0*.*77*,* 0*.*95]. In other words, in 99% of cases, the Dice values were smaller than 0.95. That is why we chose to assess bony, global and local RRs as unsuccessful if the Dice coefficients were lower than 0*.*95 or 95% of the Dice coefficients obtained without registration.

All intensity-based RR methods, except for the 1-mm local RR (*p* = 6*.*6 10^*−*2^), yielded Dice results significantly different from those obtained without registration. In addition, there was a highly significant difference between the following registration methods: 8-mm local RR vs global RR, 8-mm local RR vs bony RR, 8-mm local RR vs 1-mm local RR, 5-mm local RR vs global RR, 5-mm local RR vs bony RR, 5-mm local RR vs 1-mm local RR. Table 1 shows the Dice medians, the standard deviations (SD) and the number of failed registrations for each RR method. We obtained the best accuracy with the 5-mm and 8-mm local RRs. The two highest Dice medians, which were obtained with the local RRs with 5-mm and 8-mm margins, were close and equal to 0.816 and 0.815, respectively. The 8-mm local RR appeared to be more robust than the other intensity-based RR methods as it counted the lowest number of failed registrations (6 cases out of 115 failed, i.e., 95% of success, versus e.g., 90% of success for 5-mm local RR). When the local RR with small margins failed, it could be caused by lack of contrast and/or the frequently observed presence of (moving or not) gas pockets situated in the rectum in the vicinity of the prostate. In the following analysis, we chose to focus on the 8-mm local RR as it produced a median very close to the best one obtained with the 5-mm local RR, the lowest SD and the lowest number of failed registrations. We observed that when failing, the Dice coefficients obtained with the 8-mm local RR or the bony RR were in the same range (between 84% and 95% of the Dice coefficients without registration).

**Table 1:** **Dice results after CBCT-based setup correction (115 CT/CBCT pairs of 10 patients)**

	**Without RR**	**Manual-contour RR**	**Global RR**	**Bony RR**	**Local RR**							
		**(reference)**			**1 mm**	**3 mm**	**5 mm**	**8 mm**	**10 mm**	**12 mm**	**15 mm**	**20 mm**
Dice median	0.731	0.864	0.784	0.785	0.785	0.803	0.816	0.815	0.801	0.801	0.800	0.799
Dice SD	0.105	0.035	0.069	0.070	0.123	0.099	0.061	0.048	0.064	0.067	0.058	0.072
Failed registrations	-	-	7	8	28	21	12	6	11	12	8	9

**Figure 4:**
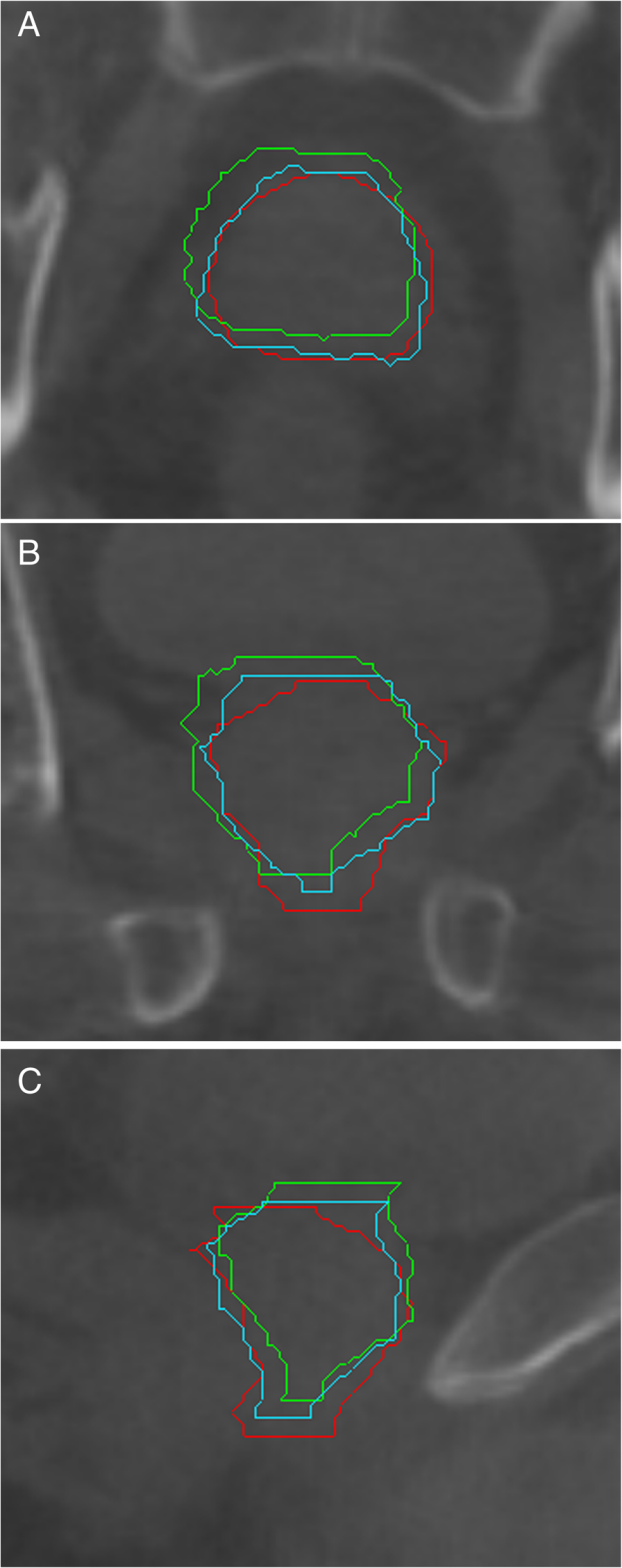
**Example of contours.** Example of (red) manual and automatic prostate contours produced by (green) bony and (blue) 8-mm local RRs displayed on top of the corresponding CBCT image in the **(A)** axial, **(B)** coronal and **(C)** sagittal planes.

Figure 4 illustrates an example of manual and automatic prostate contours produced by the bony and 8-mm local RRs displayed on top of the corresponding CBCT image. In this particular case, we obtained a Dice coefficient of 0.70 and 0.80 for the bony RR and the 8-mm local RR, respectively.

#### Bidirectional distance

We conducted the statistical analysis for the bidirectional distance in a way similar to that for the Dice coefficients. All intensity-based RR methods yielded results significantly different from those obtained without registration. We observed that the BD-based results confirmed the Dice-based results. Similarly there was a highly significant difference between the following registration methods: 8-mm local RR vs global RR, 8-mm local RR vs bony RR, 8-mm local RR vs 1-mm local RR, 5-mm local RR vs global RR, 5-mm local RR vs bony RR, and 5-mm local RR vs 1-mm local RR. Table 2 shows the BD medians and the standard deviations (SD) for each RR method. Overall we obtained the best results with the 5-mm and 8-mm local RRs in terms of BD medians (lowest, equal BD medians). However, the 8-mm local RR had the smallest SD. Thus, these results reinforced the idea that the 8-mm local RR was the most accurate method.

**Table 2:** **BD results after CBCT-based setup correction (115 CT/CBCT pairs of 10 patients)**

	**Without RR**	**Manual-contour RR**	**Global RR**	**Bony RR**	**Local RR**							
		**(reference)**			**1 mm**	**3 mm**	**5 mm**	**8 mm**	**10 mm**	**12 mm**	**15 mm**	**20 mm**
BD* median (mm)	2.86	1.48	2.18	2.17	2.20	1.95	1.84	1.84	2.01	2.01	2.04	2.10
BD* SD (mm)	1.18	0.32	0.70	0.71	1.89	1.28	0.87	0.58	0.75	0.81	0.72	0.84

*BD stands for Bidirectional Distance. It was calculated for each contour comparison by averaging the BLD values over the reference contour.

#### Impact of rectal distension on local RR quality

**Figure 5:**
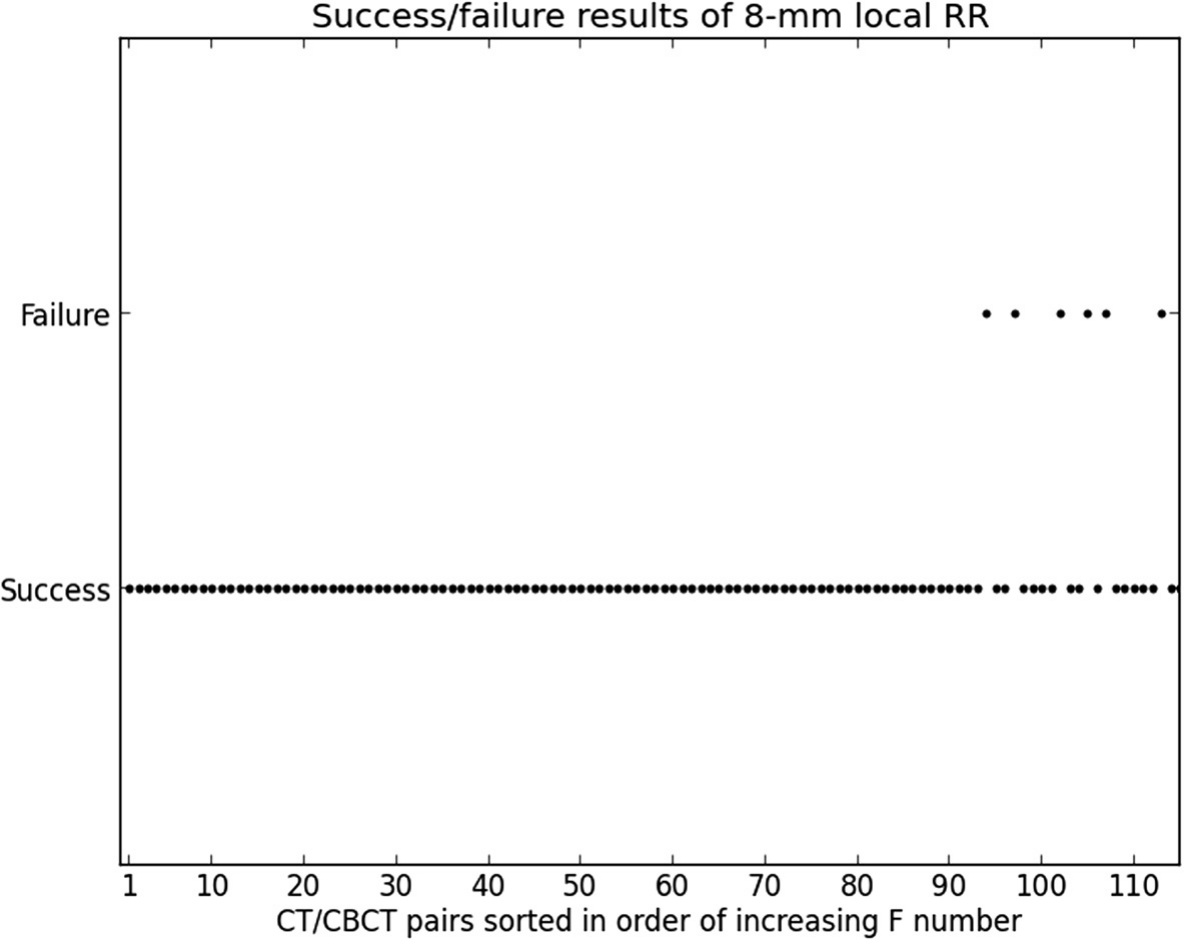
**Success failure results.** Outcome (success or failure) of 8-mm local RR w.r.t. the CT/CBCT pairs sorted in order of increasing *F* number (as defined in Equation 2).

We investigated in which cases the 8-mm local RR failed. Figure 5 illustrates the impact of the variation of rectal filling between the registered images on the 8-mm local RR quality. With our database, the *F* values obtained ranged from 0.1 to 410.7. We observed that if the *F* factor as defined in Equation 2 was lower than or equal to *F* 1 = 61*.*2, registrations were all successful (93 cases out of 115). All failed registrations (6 in total, representing almost one-third of the 22 remaining cases) appeared to have *F* values higher than *F* 2 = 147*.*6. We evaluated the performance of the 8-mm local RR with an *F* cut-off of (*F* 1 + *F* 2)*/*2 = 104*.*4 (median between *F* 1 and *F* 2). We found a sensitivity of 1 and a specificity of 0.85 with our dataset.

#### Visual assessment

Regardless of the rectum influence, the visual assessment of the segmentation quality confirmed the superiority of the 8-mm local RR over the bony RR. In total, the bony RR achieved a total cumulative score (sum of scores over all registrations) of 258 versus 291 for the 8-mm local RR. This difference was highly significant (*p* = 7*.*2 10^*−*6^). For both methods, no segmentations achieved a zero score (poor quality). The 8-mm local RR method always achieved a score better than or equal to that of the bony RR method, except for four cases (out of 115). However, in these four cases, both methods yielded segmentations acceptable for clinical use, without further correction. Table 3 indicates the numbers of segmentations that were produced by the bony and 8-mm local RRs for each score and that were considered acceptable for clinical use.

**Table 3:** **Qualitative results after CBCT-based setup correction (115 CT/CBCT pairs of 10 patients)**

**Score (per segmentation)**	**0**	**1**	**2**	**3**	**Acceptable for clinical use**
# bony RRs	0	15	57	43	107
# local 8-mm RRs	0	4	46	65	115

### Application of a replace-gas-by-tissue filter to improve registration quality

To deal with registration failures, we applied a filter to the CT and CBCT scans prior to registration to replace the intensities of the gas voxels with an intensity of tissue.

#### Dice coefficient

Table 4 shows that applying a replace-gas-by-tissue filter improved the success rate for the global RR and the local RR with a margin higher than or equal to 8 mm. For the 8-mm local RR, the success rate improved from 95% (6 failures out of 115 scan pairs) without filtering to 97% (3 failures remaining out of 115 scan pairs) when a replace-gas-by-tissue filter was applied. Out of the three pairs of scans that failed to be registered with the 8-mm local RR when a replace-gas-by-tissue filter was applied, two were successfully registered using the bony RR, and one was not successfully registered using the bony RR. For margins higher than or equal to 12 mm, the success rate improved from 90%-93% without filtering to 99% (only one failure occurred and corresponded to a pair of scans that were successfully registered with the bony RR) when filtering. In terms of success rate, when filtering, the most robust RRs were the local ones with margins higher than or equal to 12 mm. The 8-mm local RR had the highest Dice median and the lowest SD (best accuracy). Statistically, the local RRs with margins higher than 5 mm did not produce results significantly different from each other; they statistically differed from the bony RR, the global RR, and the 1-mm and 3-mm local RRs (except for the 20-mm local RR vs the global RR).

**Table 4:** **Dice results after CBCT-based setup correction (115 CT/CBCT pairs of 10 patients). Before registration, air was replaced by soft tissue in images.**

	**Without RR**	**Manual-contour RR**	**Global RR**	**Bony RR**	**Local RR**							
		**(reference)**			**1 mm**	**3 mm**	**5 mm**	**8 mm**	**10 mm**	**12 mm**	**15 mm**	**20 mm**
Dice median	0.731	0.864	0.797	0.785	0.776	0.794	0.810	0.814	0.813	0.811	0.806	0.811
Dice SD	0.105	0.035	0.062	0.070	0.113	0.123	0.074	0.045	0.052	0.046	0.052	0.055
Failed registrations	-	-	6	8	28	26	20	3	3	1	1	1

#### Bidirectional distance

Table 5 shows that for the global RR and the local RRs with margins higher than or equal to 10 mm, applying a replace-gas-by-tissue filter reduced the discrepancy between the manual and automatic contours overall (BD values lower than those obtained without filtering). Among all intensity-based RR methods, the lowest BD medians were obtained with the 5-mm, 8-mm and 10-mm local RRs. The SD of the 8-mm local RR was more than twice as low as that of the 5-mm local RR, and smaller than that of the 10-mm local RR. Therefore, in terms of BD values, the 8-mm local RR yielded more accurate results than did the other intensity-based methods.

**Table 5:** **BD results after CBCT-based setup correction (115 CT/CBCT pairs of 10 patients). Before registration, air was replaced by soft tissue in images.**

	**Without RR**	**Manual-contour RR**	**Global RR**	**Bony RR**	**Local RR**							
		**(reference)**			**1 mm**	**3 mm**	**5 mm**	**8 mm**	**10 mm**	**12 mm**	**15 mm**	**20 mm**
BD* median (mm)	2.86	1.48	2.09	2.17	2.23	2.24	1.89	1.91	1.89	1.97	2.00	1.95
BD* SD (mm)	1.18	0.32	0.64	0.71	1.90	1.69	1.08	0.49	0.59	0.55	0.61	0.66

*BD stands for Bidirectional Distance. It was calculed for each contour comparison by averaging the BLD values over the reference contour.

## Discussion

In this work, we evaluated different automatic methods for prostate localization based on intensity-based CT/CBCT RR. For the sake of simplicity, the term “local RR” was used in place of the combination of bony RR with local soft-tissue RR. On average, the execution times required by the global RR, the bony RR and the local RR were 54 s, 48 s and 1 min 10 s, respectively. Our statistical analysis showed that the most successful methods were the 5-mm and 8-mm local RRs. The success rate of the 8-mm local RR (95% of success when the air was not filtered in the rectum, and 97% of success when a replace-gas-by-tissue filter was applied) was higher than that of the 5-mm local RR and it is soundly acceptable for further implementation for clinical practice. Moreover, all automatic segmentations generated using the 8-mm local RR method (even in the unsuccessful cases) were visually considered acceptable for clinical use. In addition, we related the 8-mm local RR failures to rectal distension occurred in the vicinity of the prostate, which we estimated using an automatic method that could be easily applicable in clinical practice. We drew the conclusion that with a limited difference in rectum anatomy, an 8-mm local RR would improve the registration quality, i.e., the alignment of the registered images, and otherwise it would deteriorate the registration quality and should not be applied.

Many studies aiming to localize the prostate have been performed using daily in-room CT imaging as in [13-17]. In particular, Court *et al.* developed an automatic planning-CT/in-room-CT monomodal RR of the prostate for IGRT using a mask around the prostate. They studied the effect of the size of the registration mask using CTV expansions of 0, 3, 6 and 9 mm, and showed that the optimum expansion was 3 mm [15]. However, they quantitavely analyzed only 28 image sets from 2 patients and considered only translations. They also showed that in the presence of air in the rectum, filtering the air out of the registration mask produced better results. Later on, Smitsmans *et al.* conducted a study similar to ours with monomodal CT/CT RR with 19 patients and 8-13 repeat CT scans per patient, and they found the optimum margin was 5 mm [17]. They also showed that applying a filter before registration that removed gas from the registration mask or that replaced rectal gas by soft tissue improved the results. To evaluate the results, they compared the results of intensity-based registration to that of contour registration (in terms of volume overlap, and mean and SD of the differences for each rotation and translation axis for successful registrations), the latter being used as a reference (contours were drawn manually on each planning and repeat CT scan).

Daily in-room CBCT imaging for prostate cancer was used in [20-29] but very few studies localized the prostate in a completely automatic way. In [20,21], manual CT/CBCT soft-tissue RR and alignments of implanted fiducials using orthogonal kV or MV portal images and CBCT scans were compared to target the prostate. In [22], commercial software using an automatic intensity-based RR algorithm was used to align implanted *I*^125^ seeds. Without proposing a strategy to automatically localize the prostate on the CBCT scan, authors in [23] measured the residual setup error for prostate cancer patients after online CBCT-based setup correction using three radiopaque markers made of high-winding coils in the prostate and prostate contours drawn manually on the CT and CBCT scans. In [24], commercial software was used to perform an automatic intensity-based soft-tissue RR.

Kim *et al.* tested the effect of different similarity metrics and expansions (ranging from 0 to 10 mm) for the prostate registration mask on automatic CT/CBCT registration quality [25]. They removed the gas and the pelvic bone from the registration mask. They found that expansion margins of 4-10 mm were equally successful. The registration accuracy was assessed using one natural prostate calcification in images (not a global measure) as well as qualitative visual evaluation. A minutely detailed inspection of their results showed us that among all the margins they had tested (from 0 mm to 10 mm), they had obtained the most accurate results with the 8-mm margin (mean *±* SD = 1*.*5 *±* 0*.*7 mm, but more particularly the lowest maximum calcification mismatch error (3.6 mm)), which is in agreement with our results.

Smitsmans *et al.* automatically localized the prostate on CBCT scans using their above-mentioned algorithm with a 5-mm margin (assuming that the optimal margin found for CT/CT registration will be optimal for CT/CBCT registration as well) and a “replace-gas-by-tissue” filter [26]. They also removed the pelvic bone and the prostate calcifications close to the border (subject to movement) from the registration mask. To evaluate the results, a visual inspection was performed (a registration was assessed as successful if the prostate could fit within the manual CT contours expanded by 3.6 mm and overlaid on the registered CBCT scan). Registration errors of calcification mismatch were determined for patients with calcifications within the prostate, and only for successful registrations. In our study, we showed that when we filtered the air in the rectum as done in [17], we obtained an optimum margin of 8 mm (best accuracy in terms of Dice and BD values among all methods and a success rate of 97% better than the 83% success rate we obtained with the 5-mm margin). That said, margins between 8 mm and 15 mm yielded close results in terms of Dice median, SD, success rate and from a statistical point of view (as for the 20-mm local RR, it was not found to be significantly different from the global RR). The fact that we do not find the same optimum margin can stem from the nature of the images being registered (monomodal CT-to-CT registration versus multimodal CT-to-CBCT registration) and the nature of the validation (e.g., different definitions of registration success). In [26], authors also reported that the CT/CBCT local RR with a 5-mm margin mainly failed because of streaks in the CBCT scans caused by moving gas pockets in the rectum but they did not propose an automatic and quantitative way to predict failures. In our study, we proposed an automatic method to estimate rectal distension to predict registration failures, using only the manual CT contours with the gray-value CT and CBCT images. The manual CBCT contours were not needed.

In this work, we did not study the inter/intra-observer variability in manual delineation, hence nor the amount of Dice uncertainty due to this variability. This can be investigated in a future study. White *et al.* have previously determined the inter-observer variability of defining the prostate on CBCT images in terms of variations in volume, center of mass, prostate boundary and consequent isocenter placement [39]. Weiss *et al.* have also analyzed inter/intra-observer contouring variations using standard deviation and average volume calculations [40]. However, none of these studies calculated Dice coefficients of volumes delineated by different observers.

The method we proposed in this paper to assess rectal distension could be applied in an adaptive radiotherapy approach. A future study could aim at assessing the impact of rectal distension on dose target coverage and organ-at-risk exposure. This could allow one to determine a cut-off value for rectal distension beyond which treatment replanning would become necessary.

## Conclusions

With this study, we aimed to provide guidance for good practices in the use of CT-to-CBCT RR for prostate position verification and correction. We recommend to start with a bony RR. The next step is to determine whether a local RR with an 8-mm margin can be performed on top of the bony RR to improve upon registration quality. To do so, the user should estimate rectal distension occurred in the vicinity of the prostate between the planning CT scan and the treatment CBCT scan. In this paper, we propose a method to conduct such an evaluation that is easily applicable in clinical practice and automatic using only the manual CT contours and requiring calculations of mean intensities in the prostate and in the portion of the rectum included in the registration mask in both images after bony RR. If the difference in rectum anatomy is limited, the 8-mm local RR will improve registration quality and prostate targeting. If not, the 8-mm local RR may deteriorate registration quality and hence it should not be applied. We highly recommend that the user should always visually assess the final registration quality, particularly when a local RR is applied.

## Endnotes

^a^freely available at www.itk.org.

^b^freely available at www.creatis.insa-lyon.fr/rio/vv.
